# Periodontal Tissue as a Biomaterial for Hard-Tissue Regeneration following *bmp-2* Gene Transfer

**DOI:** 10.3390/ma15030993

**Published:** 2022-01-27

**Authors:** Mariko Yamamoto Kawai, Ryosuke Ozasa, Takuya Ishimoto, Takayoshi Nakano, Hiromitsu Yamamoto, Marina Kashiwagi, Shigeki Yamanaka, Kazumasa Nakao, Hiroki Maruyama, Kazuhisa Bessho, Kiyoshi Ohura

**Affiliations:** 1 Department of Welfare, Kansai Women’s College, Osaka 582-0026, Japan; 2 Department of Oral and Maxillofacial Surgery, Graduate School of Medicine, Kyoto University, Kyoto 606-8507, Japan; yamamoto.hiromitsu.33u@st.kyoto-u.ac.jp (H.Y.); kashiwagim@kuhp.kyotou.ac.jp (M.K.); yama0821@kuhp.kyotou.ac.jp (S.Y.); knakao@kuhp.kyoto-u.ac.jp (K.N.); bes@kuhp.kyoto-u.ac.jp (K.B.); 3 Division of Materials and Manufacturing Science, Graduate School of Engineering, Osaka University, Osaka 565-0871, Japan; ozasa@mat.eng.osaka-u.ac.jp (R.O.); ishimoto@sus.u-toyama.ac.jp (T.I.); nakano@mat.eng.osaka-u.ac.jp (T.N.); 4 Center for Aluminum and Advanced Materials Research and International Collaboration, School of Sustainable Design, University of Toyama, Toyama 930-8555, Japan; 5 Department of Clinical Nephroscience, Graduate School of Medicine and Dental Sciences, Niigata University, Niigata 951-8501, Japan; hirokim@med.niigata-u.ac.jp; 6 Department of Nursing, Taisei Gakuin University, Osaka 587-8555, Japan; k-ooura@tgu.ac.jp

**Keywords:** periodontal tissue, BMP, gene therapy, collagen micro-arrangement, bone quality

## Abstract

The application of periodontal tissue in regenerative medicine has gained increasing interest since it has a high potential to induce hard-tissue regeneration, and is easy to handle and graft to other areas of the oral cavity or tissues. Additionally, bone morphogenetic protein-2 (BMP-2) has a high potential to induce the differentiation of mesenchymal stem cells into osteogenic cells. We previously developed a system for a gene transfer to the periodontal tissues in animal models. In this study, we aimed to reveal the potential and efficiency of periodontal tissue as a biomaterial for hard-tissue regeneration following a *bmp-2* gene transfer. A non-viral expression vector carrying *bmp-2* was injected into the palate of the periodontal tissues of Wistar rats, followed by electroporation. The periodontal tissues were analyzed through bone morphometric analyses, including mineral apposition rate (MAR) determination and collagen micro-arrangement, which is a bone quality parameter, before and after a gene transfer. The MAR was significantly higher 3–6 d after the gene transfer than that before the gene transfer. Collagen orientation was normally maintained even after the *bmp-2* gene transfer, suggesting that the *bmp-2* gene transfer has no adverse effects on bone quality. Our results suggest that periodontal tissue electroporated with *bmp-2* could be a novel biomaterial candidate for hard-tissue regeneration therapy.

## 1. Introduction

Periodontal tissue consists of the gingiva, periodontal ligament (PDL), and alveolar bone. Mesenchymal stem cells (MSCs) occur in periodontal tissue, including the PDL [1,2,3]. MSCs are a heterogeneous population derived from mesenchymal tissue and have the functional capacity to differentiate into bone, cartilage, and adipose cells in vitro [4,5]. PDL is also an essential factor for dental implant therapy; it is especially related to microbial contamination [6]. In addition to self-regeneration, periodontal tissue has a high potential to induce hard-tissue regeneration, such as tooth and bone tissues [1,2,3,4,5,7]. In regenerative therapy, the handling and grafting of periodontal tissue to other areas of the oral cavity or other tissues is a simple process for surgeons [8,9]. 

Bone morphogenetic protein (BMP) has a high potential to differentiate MSCs into osteogenic cells [10,11,12,13,14]. However, the application of BMP-based biomaterials for bone regeneration has limitations [15,16,17,18]. For example, BMP-2 requires a high-quality purification process for human tissue application, and it causes the biomaterials with BMP-2 high cost [15,16,17,18]. Adequate scaffolds, such as apatite or collagen, are required to retain BMP-2 at the target site because only BMP-2 is released and spreads immediately after injection [15,16,17,18,19,20]. Recombinant BMP-2 has been utilized for bone regeneration therapy [21,22,23,24]. However, long-term and high-dose BMP-2 treatment leads to osteoclastogenesis owing to negative feedback [25].

We previously developed a gene transfer system for ectopic bone formation in the skeletal muscles of rats [26,27,28,29]. For our gene transfer system, we constructed non-viral vectors: pCAGGS-*bmp-2*, which produces human BMP-2 homodimers [26] and pCAGGS-*bmp-2/7*, which produces human BMP-2/7 heterodimers [29]. pCAGGS can express external genes temporarily at the injection site [30]. Therefore, we considered that our pCAGGS constructs could be applied to the regeneration of periodontal tissues, such as alveolar bone.

Of these two constructs, BMP-2/7 heterodimer is known to have a greater potential to induce ectopic bone formation [29,31]. Therefore, at first, we tried applying pCAGGS-*bmp-2/7* for alveolar bone induction in rats; we successfully induced the formation of new alveolar bone after the transfer of this gene expression vector to periodontal tissue [32]. However, BMP-2 also has a high potential to induce the differentiation of mesenchymal stem cells to osteogenic cells [29]. Moreover, recombinant human BMP-2 has already been used clinically [33,34]. Therefore, in this study, we applied our pCAGGS-*bmp-2* construct for alveolar bone regeneration by transferring it to the periodontal tissues of rats.

To verify the safety and efficacy of the new biomaterials, it is essential to evaluate the quality of bone formed. Bone tissue has highly organized microstructures composed of oriented collagen and apatite crystallites, which play essential roles in bone mechanical functions [35,36]. However, obtaining an appropriate anisotropic collagen/apatite microstructure during the process of bone regeneration remains a great challenge [37] because collagen/apatite orientation is regulated by complex biological events that occur in an in vivo environment. In this study, we attempted to reveal the potential and efficiency of periodontal tissue as a biomaterial for hard-tissue regeneration following a *bmp-2* gene transfer by in vivo electroporation.

## 2. Materials and Methods

### 2.1. Preparation of bmp-2 Gene Expression Plasmids

The pCAGGS plasmid was donated by Prof. J. Miyazaki of Osaka University. The construction of the pCAGGS-*bmp-2* plasmid has been described previously [26,29]. We prepared the plasmid vectors using a Qiagen EndoFree Plasmid Giga Kit (Qiagen, Hilden, Germany) using *Escherichia coli* DH5 culture.

### 2.2. Gene Transfer to Rat Periodontal Tissue

Nine-week-old male Wistar rats were purchased from Kurea (Osaka, Japan) and maintained under specific pathogen-free conditions in our animal facility. Rats were allocated into the control group without *bmp-2* and the *bmp-2* gene-injected groups (*n* = 3 per group). Rats were anesthetized by the subcutaneous injection of medetomidine hydrochloride (0.5 mg/kg, Dormitor, Zenoaq, Fukushima, Japan), midazolam (4 mg/kg, Sandoz, Basel, Switzerland), and butorphanol (5 mg/kg, Beltfoul; Meiji, Tokyo, Japan). Each plasmid (0.5 µg/µL) was injected into the palatal region of periodontal tissue in the first molar of the maxilla targeting the periosteal and periodontal ligament. Electroporation (conditions: 32 pulses of 50 V for 50 ms) was delivered directly to the tissue via needle-type electrodes (Neppa Gene, Tokyo, Japan) using a portable electroporation device (Genepulser; Ohta, Okayama, Japan). All procedures were approved by the Animal Care and Use Committee of Osaka Dental University (Approval number: 19-02016).

### 2.3. Bone Double Staining

**Figure 1:**
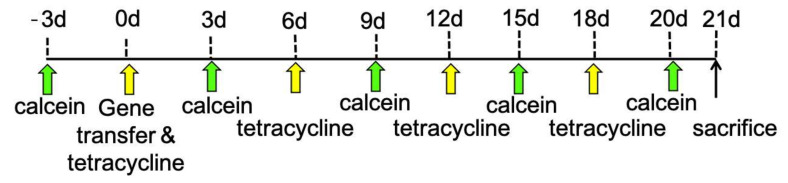
Scheme of injections for bone double staining with calcein and tetracycline hydrochloride. We first injected calcein intraperitoneally. Three days later, we injected tetracycline hydrochloride and, simultaneously, gene transfer was performed. We injected calcein again 3, 9, 15, and 20 days after gene transfer, and tetracycline 6, 12, and 18 days after gene transfer. Finally, the rats were euthanized 21 days after gene transfer.

Nine-week-old male Wistar rats (*n* = 3 per group) were injected intraperitoneally with calcein (10 mg/kg) 3 days before gene transfer. Gene transfer was performed after the intraperitoneal injection of tetracycline hydrochloride (30 mg/kg). We injected calcein 3, 9, 15, and 20 days after gene transfer, and tetracycline 6, 12, and 18 days after gene transfer (Figure 1). Rats were euthanized with an overdose of sodium pentobarbital 21 days after gene transfer. The maxillary regions of the rats were dissected and fixed with 70% ethanol for 8 days, stained with Villanueva Osteochrome Bone stain for 10 days, dehydrated with increasing concentrations of ethanol, and embedded in methyl methacrylate with decalcification [34].

### 2.4. Bone Morphometric Analyses

After polymerization, we obtained 10-µm frontal sections from the mesiolingual center of the upper first and second molars. We observed the sections by fluorescence microscopy under UV visible irradiation to detect tetracycline (364 nm) and calcein (477 nm) staining. The distance between the calcein and tetracycline signal was measured vertically in 10 different points within the region affected by gene transfer using a Histometry RT Camera (System Supply, Tokyo, Japan). The 10 selected points were those in which signals of calcein and tetracycline were lined horizontally, and the surface of alveolar bones was smooth. Statistical comparisons of MAR were performed between the data at each specific time point and that before gene transfer as a reference data with no effects of gene transfer, using unpaired two-tailed *t*-tests.

### 2.5. Collagen Orientation Analyses

To investigate the effects of *bmp-2* gene transfer on bone quality in the alveolar bone around the upper first molar, this study focused on three bone regions from rats with or without *bmp-2* gene injection as follows: (i) control group with sham operation (no *bmp-2* injection); (ii) *bmp-2*-injected group; and (iii) opposite side of the *bmp-2*-injected group without treatment. This study used groups (i) and (iii) as comparison groups for the *bmp2*-injected group. The non-decalcified bone sections after bone morphometric analyses were used in this analysis. Bone sections were observed using a two-dimensional birefringence measurement system (WPA-micro; Photonic Lattice, Miyagi, Japan) attached to an upright microscope (BX60; Olympus, Tokyo, Japan). Birefringence analysis of collagen was performed using WPA-VIEW software (v2.4.2.9; Photonic Lattice), as previously described [35,36]. The orientation order parameter *f*_θ_ was calculated based on the angle distribution of the collagen against the reference direction. Here, *f*_θ_ is a value ranging from −1 (collagen perfectly aligned perpendicular to the reference direction) to 1 (collagen perfectly aligned parallel to the reference direction). Statistical analyses were performed using a one-way analysis of variance followed by post-hoc Tukey’s honestly significant difference comparisons.

## 3. Results

### 3.1. Bone Labeling and Mineral Apposition Rate (MAR)

**Figure 2:**
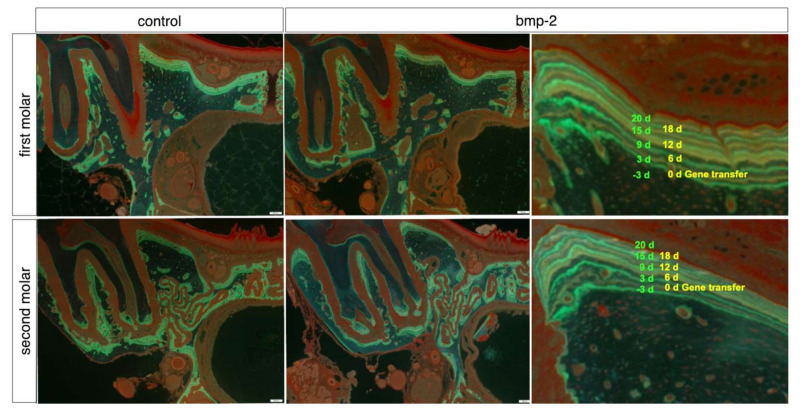
Histomorphometric analysis by bone double staining with calcein (green line) and tetracycline (yellow line) in the alveolar bone of the first or second molars. As a control group: alveolar bone in the first molar electroporated with pCAGGS, or alveolar bone in the second molar of those electroporated with pCAGGS in the first molar. As *bmp-2* group: alveolar bone in the first molar electroporated with pCAGGS-*bmp-2*, or alveolar bone in the second molar of those electroporated with pCAGGS-*bmp-2* in the first molar. The nine lines are labeled by calcein and tetracycline. Scale bar, 200 μm.

**Figure 3:**
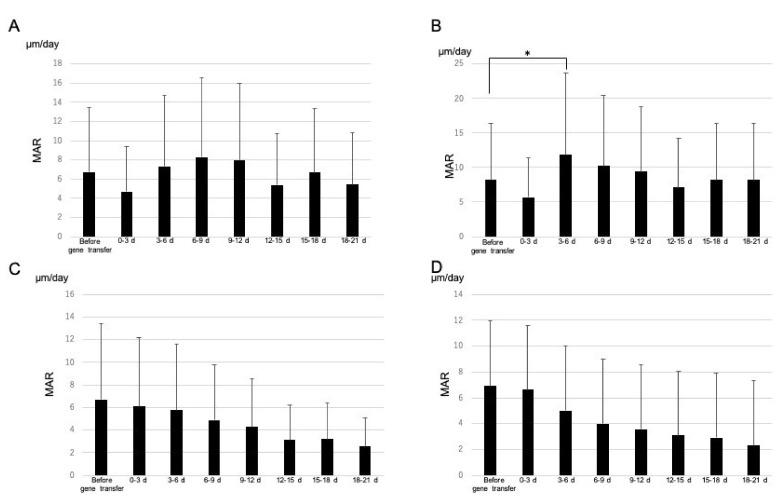
(**A**) MAR of alveolar bone in the first molar electroporated with pCAGGS. (**B**) MAR of alveolar bone in the first molar electroporated with pCAGGS-*bmp-2*. (**C**) MAR of alveolar bone in the second molar following transfer of pCAGGS to the first molar. (**D**) MAR of alveolar bone in the second molar following transfer of pCAGGS-*bmp-2* to the first molar. We set basal MAR values based on recordings made 0–3 days before gene transfer. Each bar represents population standard deviation of the mean. * *p* < 0.05 for MAR vs. basal MAR values obtained from unpaired two-tailed *t*-tests.

We found nine sites with double staining in the alveolar bones of the first and second molars (Figure 2). We set the baseline level as the MAR value 3 days before the gene transfer; MAR values for the first molar or second molar (Figure 2) after the gene transfer were then compared with the baseline level. These values were assessed every 3 days from 0 to 20 days. In the empty plasmid vector group, we could not find any significant differences in MAR values for the first and second molars (Figure 3A,C). However, we found a significant difference in MAR values for the first molar between 3–6 days after the gene transfer and before the gene transfer (Figure 3B). In comparison, the MAR values for the second molar after the gene transfer did not differ significantly from those before the gene transfer (Figure 3D).

### 3.2. Collagen Fiber Orientation

**Figure 4:**
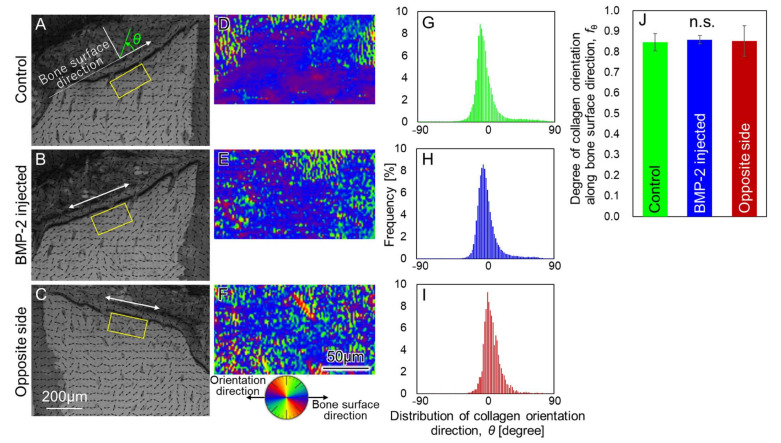
Assessment of bone quality based on collagen orientation. (**A**–**C**) Collagen orientation map in the frontal section. Arrows indicate the preferential direction of collagen orientation. (**D**–**F**) High-resolution mapping of collagen orientation direction distribution in the squared regions of (**A**–**C**). The blue color indicates the preferential orientation of collagen along the bone surface direction. (**G**–**I**) Quantified collagen orientation distribution in the squared regions of (**A**–**C**). The bone surface direction coincides with 0 degrees (**J**) Degree of collagen orientation along the bone surface direction. There was no statistical dif-ference in bone quality among the groups, suggesting that bone with sound quality was formed in the bmp-2 injected group. N.S.: not significant.

Figure 4 shows collagen orientation in terms of alveolar bone quality around the upper first molar. Birefringence analysis revealed a two-dimensional distribution of collagen orientation direction. The color map (Figure 4D–F) and histogram (Figure 4G–I), that are drawn using the bone surface direction as the reference axis, clearly showed that collagen was aligned approximately parallel to the bone surface direction in the frontal plane. The degree of collagen orientation along the bone surface direction was not significantly different between experimental groups (Figure 4G). This indicated that the *bmp-2* injection did not result in a deterioration of the collagen micro-arrangement. The findings obtained here suggest that the *bmp-2* gene transfer has no adverse effect on the collagen microstructure during bone formation.

## 4. Discussion

In this study, we evaluated the potential of periodontal tissue to regenerate alveolar bone after a *bmp-2* gene transfer via in vivo electroporation. In our previous study, we induced new alveolar bone growth in original bone tissues (similar to mini-modeling), after transferring pCAGGS-*bmp*-2/7 to periodontal tissues [32]. On the other hand, we could not observe new alveolar bone growth in the present study (data not shown). However, when we examined alveolar bone regeneration for 3 weeks after the *bmp-2* gene transfer, via the measurement of MAR after bone double staining with calcein and tetracycline. MAR is one of the parameters of bone morphometric analyses [38]. Bone morphometric analyses could catch the chronological change in bone formation or regeneration [39,40]. In this study, we found that MAR was significantly higher 3–6 days after the *bmp-2* gene transfer than before the gene transfer. This suggests that the periodontal tissues transferred with pCAGGS-*bmp-2* have the potential to increase alveolar bone regeneration, even though no new alveolar bone was observed. 

In contrast, there were no significant differences in MAR before and 3 weeks after the gene transfer in the control group. We did not find any significant differences in MAR between 0–3 days after the *bmp-2* gene transfer and before the gene transfer. In our previous study, there was also no significant difference in MAR for up to 3 days after the *bmp-2/7* gene transfer compared with the *bmp-2/7* and *lacZ* gene transfer [32]. We assumed that it might take 3 days for the exogenous BMP-2 derived from the *bmp-2* gene transfer to accumulate in the periodontal tissues and affect the MAR of the alveolar bone. We need more detailed studies to elucidate this assumption, such as time-course studies for exogenous BMP-2 derived from a gene transfer.

However, it is a concern that a *bmp-2* gene transfer, which increases MAR, would have adverse effects on bone quality, such as the bone matrix (collagen and apatite) micro-arrangement, because MAR is negatively related to the preferential orientation of collagen/apatite [41,42,43]. The effect of a gene transfer on the anisotropic micro-arrangement of the bone matrix in regenerated bone is unknown, in spite of the importance of the bone matrix quality for the mechanical function of bone. Impaired bone mechanical function, owing to disturbed collagen/apatite orientation, has been shown in various bone disorders, such as osteoporosis [44], osteopetrosis [45], cancer metastasis [46], and chronic kidney disease [47]. Thus, the maintenance of an intact collagen/apatite orientation during bone regeneration is imperative for realizing proper bone mechanical function. Regenerative therapy with a *bmp-2* gene transfer did not result in the deterioration of collagen orientation, as revealed in this study. Thus, we propose that a *bmp-2* gene transfer, which is expected to increase MAR with no adverse effects on bone quality, could be a beneficial technique for bone regenerative medicine.

Gene therapy has been used to treat general disease rather than local disease [48,49,50]. In the gene transfer system developed in this study, with a nonviral plasmid vector and in vivo electroporation, we could control the gene transfer area because electrodes played an important role in restricting, as external genes could be transferred only in the area stimulated by electricity [29]. This explains why there were no significant differences in the MARs of the alveolar bone in the second molar from basal levels before the gene transfer. Collectively, these results suggest that periodontal tissues after a *bmp-2* gene transfer could serve as novel biomaterials for bone regeneration. However, the amount of the *bmp-2* gene transfer and the transfer scheme need to be optimized to further promote osteogenesis, which will be a future challenge. Furthermore, future analysis might include examining regeneration beyond the alveolar bone surrounding periodontal tissues by grafting periodontal tissue transferred with *bmp-2* to defecated bone tissue, and inducing bone regeneration by other methods, such as µMRI or µCT [51], not only bone morphometric analyses including MAR measurement.

## 5. Conclusions

The periodontal tissues *bmp-2* gene, transferred by non-viral plasmid vectors and in vivo electroporation, had a high potential to increase MAR from 3 to 6 days after the gene transfer. Despite the elevated MAR, bone quality characterized by collagen orientation remained normal. Thus, periodontal tissue after a *bmp-2* gene transfer presents as a novel biomaterial candidate for hard tissue regeneration therapy, which enhances bone formation without adverse effects in bone quality, without also requiring artificial scaffolds and expensive proteins.
